# Empirical Relationships between Problematic Alcohol Use and a Problematic Use of Video Games, Social Media and the Internet and Their Associations to Mental Health in Adolescence

**DOI:** 10.3390/ijerph17176098

**Published:** 2020-08-21

**Authors:** Lutz Wartberg, Rudolf Kammerl

**Affiliations:** 1 Department Psychology, Faculty of Life Sciences, MSH Medical School Hamburg, 20457 Hamburg, Germany; lutz.wartberg@medicalschool-hamburg.de; 2 Department of Education, Chair for Pedagogy with a Focus on Media Education, Friedrich-Alexander-University Erlangen-Nuremberg, 90478 Nuremberg, Germany

**Keywords:** Internet addiction, pathological Internet use, Internet gaming disorder, gaming disorder, social networking site addiction, social media addiction, Facebook addiction, problem drinking, alcohol, adolescent

## Abstract

Adolescents frequently show risky behavior, and these problematic behavior patterns often do not occur in isolation, but together. Problematic alcohol use is widespread among youth, as is problematic use of the Internet and of specific online applications (video games or social media). However, there is still a lack of findings for minors regarding the relations between these behavioral patterns (particularly between problematic alcohol use and problematic gaming or problematic social media use). Standardized instruments were used to survey problematic alcohol use, problematic gaming, problematic social media use, problematic Internet use and mental health among 633 adolescents (mean age: 15.79 years). Bivariate correlation and multivariable linear regression analyses were conducted. The correlation analyses showed statistically significant positive bivariate relationships between all four behavioral patterns each. Antisocial behavior was related to all problematic behavioral patterns. Whereas, emotional distress, self-esteem problems and hyperactivity/inattention were associated with substance-unrelated problematic behavior patterns only. Anger control problems were related to problematic alcohol use and problematic gaming. In adolescence, the findings revealed small effect sizes between substance-related and substance-unrelated problematic behavior patterns, but moderate to large effect sizes within substance-unrelated behavioral patterns. Similarities and differences were found in the relations between the behavioral patterns and mental health.

## 1. Introduction 

In adolescence, new developmental tasks can pose great challenges and psychological stress for minors [1]. In addition to effective problem-solving strategies, dysfunctional stress management strategies (e.g., use of psychotropic substances) are often used to cope with these developmental tasks [1], which can also serve the striving for independence (e.g., from parents) or the search for identity [2]. According to the Problem-Behavior Theory of Jessor [3], these dysfunctional stress management strategies or problematic behavior patterns often do not occur in isolation, but together. Problematic alcohol use (often referred to as problem drinking) is explicitly mentioned by Jessor [3] (p. 602). The extensive technological changes in recent years and decades call for the concept of Problem-Behavior Theory to be expanded in content. Some authors (e.g., De Leo & Wulfert [4]) now also include a problematic use of the Internet or its applications in this context. Considering the widespread occurrence of substance-related problematic behavior patterns and problematic use of digital media in adolescence, it seems quite relevant to examine whether there are empirical relationships. Initial studies (e.g., [5]) have conducted a combined investigation of substance-related problem behavior (e.g., problematic alcohol use) and problem behavior unrelated to substance use (e.g., problematic Internet use or problematic use of specific online applications). Another important approach specifically to explain Internet-use disorders is the Interaction of Person-Affect-Cognition-Execution (I-PACE) model by Brand et al. [6]. In the I-PACE model specific Internet-use disorders “…are considered to be the consequence of interactions between predisposing factors” (e.g., biopsychological constitution, psychopathology, personality, social cognitions and specific motives for using), “…coping styles and Internet-related cognitive biases…” and ”…affective and cognitive responses to situational triggers in combination with reduced executive functioning” (p. 252).

The increasing relevance of problem behavior unrelated to substance use was demonstrated by the American Psychiatric Association (APA) with the inclusion of “Internet Gaming Disorder” in the appendix of the DSM-5 [7] (as a condition for which further studies are required), and by the World Health Organization (WHO) with the inclusion of “Gaming Disorder” in the ICD-11 [8]. In addition to use of video games (in the following referred to as gaming), the use of social media is often mentioned as another Internet application with potential for problematic use by adolescents (e.g., [9]). The collective term “social media” includes the use of websites of social networks and messengers as well as blogs, etc. [10]. In addition, an increasing number of studies is examining problematic use of the Internet on a more general level [11]. According to available empirical findings, it seems appropriate to differentiate between general problematic Internet use and the use of specific applications (such as online gaming, in cross-section: (e.g., [12]), in longitudinal section: [13]). 

As an important substance-related behavior pattern, problematic consumption of alcohol and its consequences in adolescence have been a relevant aspect of public health efforts and scientific research for a long time (in Germany, for example, an increase in the age of first alcohol use from 14.1 years in 2004 to 15.0 years in 2018 has been observed [14]). Indeed, alcohol use among adolescents remains widespread in many countries. Following the European School Survey Project on Alcohol and Other Drugs (ESPAD) risky patterns of alcohol consumption are still highly prevalent in youth. In 1995, in the first ESPAD study, 36% of the examined European students reported heavy episodic drinking. In 2015, in the last ESPAD investigation, the observed prevalence for heavy episodic drinking was 35% [15]. Besides, a substantial percentage of youth already shows a problematic alcohol use. In Germany (where the present study was conducted), the one-year prevalence estimation for problematic alcohol use in a representative sample of adolescents was 5.0% [16].

In addition to substance-related problem behavior (especially alcohol consumption), the relevance of problem behavior unrelated to substance use (such as problematic Internet use, problematic gaming, and problematic social media use) has often been discussed and empirically investigated especially in adolescence. Surveys of representative samples of youth show high prevalence values for problematic Internet use, problematic gaming, and problematic social media use. Based on latent profile and latent class analyses in representative samples, prevalence estimates between 3.2% and 4.7% were observed for problematic Internet use in German minors [17,18,19]. For problematic gaming, in representative samples prevalence values of 2.6% among Slovenian students and 3.5% in German adolescents were reported [20,21]. Bányai et al. [22], investigated a representative sample of Hungarian students, conducted a latent profile analysis and obtained a prevalence estimate of 4.5% for problematic social media use. At present, it is still largely unclear how often problematic alcohol use and problematic use of the Internet use or specific online applications occur combined in adolescence.

According to the Problem-Behavior Theory of Jessor [3], associations between problematic alcohol and problematic use of the Internet or specific online applications seem not unlikely, but relatively few empirical findings on these relations have been published. Relationships between problematic Internet use and general substance use in adolescents have been investigated more frequently (see for example the systematic review of Lanthier-Labonté et al. [23]). However, it is important to distinguish between general alcohol consumption (which in Germany occurs among a majority of minors) and problematic alcohol use in adolescence (which only affects a minority, but which clearly can have serious consequences, see for instance the review of McCambridge et al. [24]).

In the few available studies, relations between problematic alcohol use and problematic Internet use in adolescence were usually examined in cross-sectional surveys. Pallanti et al. [25] observed a statistically significant positive correlation (0.38) between problematic alcohol use and problematic Internet use among Italian youth (average age: 16.67 years). In the study by Ha et al. [26], problematic alcohol use in South Korean adolescents (mean age: 15.8 years) did not occur more frequently in youth with problematic Internet use than among those without problematic Internet use. In contrast, Ko et al. [5] reported that problematic alcohol use occurred more often in Taiwanese adolescents with problematic Internet use than without problematic Internet use (average age of the sample was 16.26 years). Golpe et al. [27] found a statistically significant positive correlation (0.36) between problematic alcohol use and problematic Internet use among Spanish youth (mean age: 14.52 years). Rial et al. [28] obtained more problematic alcohol use among Spanish minors (average age: 14.41 years) with problematic Internet use than among adolescents without problematic Internet use. In addition, in a longitudinal study Gámez-Guadix et al. [29] showed that negative consequences of problematic Internet use predicted an increase in problematic alcohol use in Spanish minors (mean age: 14.92 years) six months later.

For relations between problematic alcohol use and problematic gaming there are so far only findings from studies with adults. Na et al. [30] observed relatively often (in 165 of the 1819 surveyed 20- to 49-year olds) a comorbid occurrence of problematic alcohol use and problematic gaming in South Korea and this comorbidity group showed more severe psychopathological impairments compared to the adults affected by only one of the two problem behavioral patterns. In contrast, the survey by Erevik et al. [31] showed a negative association between “high-level gaming” and problematic alcohol use among Norwegian university students (average age: 25.8 years). Problematic gaming was a protective factor against problematic alcohol use in this investigation. For relations between problematic alcohol use and problematic social media use only one result (also in adults) has been published so far [32]. Lyvers et al. [32] obtained a statistically significant positive correlation (0.42) between problematic alcohol use and problematic social media use in Australian adults (mean age: 26.1 years).

Furthermore, in both the I-PACE model (“psychopathology”) [6] and the Problem-Behavior Theory (e.g., “low self-esteem”) [3] associations to mental health were also mentioned. Problematic alcohol use (e.g., [33]), problematic gaming (e.g., [9]), problematic social media use (e.g., [9]) and problematic Internet use (e.g., [12]) are each associated with a higher psychopathological burden, but there are very few studies that have examined several of these patterns and their links to mental health in one sample (e.g., [33]).

To sum up, empirical evidence predominantly suggests relations between problematic alcohol use and problematic Internet use in adolescence [5,25,27,28,29]. Whether such associations exist between problematic alcohol use and problematic gaming or problematic social media use is currently unclear for youth. Accordingly, the present study is the first to investigate empirically associations between problematic alcohol use and problematic gaming and problematic social media use in adolescence. Furthermore, we wanted to explore and compare associations between problematic alcohol use, problematic gaming, problematic social media use, problematic Internet use and different aspects of mental health.

## 2. Materials and Methods

### 2.1. Research Questions

In the present study the following research questions (RQs) were examined:

RQ1 What is the relationship between problematic alcohol use and problematic gaming in adolescents?
RQ2 What is the relationship between problematic alcohol use and problematic social media use in adolescents?
RQ3 What is the relationship between problematic alcohol use and problematic Internet use in adolescents?
RQ4 What are the relationships between problematic alcohol use, problematic gaming, problematic social media use, problematic Internet use and different aspects of mental health?

### 2.2. Procedure

Data collection was conducted in Germany within the framework of the VEIF project in accordance with the Declaration of Helsinki. The Ethics Committee of the German Educational Research Association (Deutsche Gesellschaft für Erziehungswissenschaft, DGfE) approved the proceedings (approval number: 01/2018/DGfE). Informed consent was obtained both from the surveyed adolescents and from one of their parents. The findings reported below are based on nation-wide data collected in the first quarter of 2019 by a market research institute. The VEIF project is a longitudinal study in which data had previously been collected at annual intervals in the first quarter 2016, in the first quarter 2017 and in the first quarter 2018. At the time of the first data collection (2016), youth were aged between 12 and 14, and in 2019 between 14 and 17. As formulated in the research questions, relations between problematic alcohol use and problematic use of the Internet and specific online applications should be investigated. For the first time in the 2019 data collection, due to the increased age of the sample, a substantial and sufficient number of adolescents (n = 57) reported problematic alcohol use. The data collection was carried out in 2019 by 134 interviewers directly at the families’ homes. Using computer-assisted personal interviewing (CAPI), one adolescent and one parent (dyad) were surveyed each. For the dyads, the parent was examined first and afterwards the adolescent. In the VEIF project, a sample with an increased risk of problematic use of digital media compared to the general population is examined. For this purpose, an oversampling of youth with an increased risk of problematic use of digital media was carried out before the first data collection in 2016 (a more detailed description of the study design and the recruitment process conducted at the beginning of the VEIF project can be found at Wartberg et al. [34]).

### 2.3. Measures

Problematic alcohol use in the last 12 months was measured with the Alcohol Use Disorders Identification Test-Consumption (AUDIT-C, Bush et al. [35]). We used the German version by Lampert and Kuntz [36]. The screening questionnaire comprises three items. In the first AUDIT-C question, the adolescents were surveyed how often they have a drink containing alcohol (five-step response format: 0 = “never”, 1 = “monthly or less”, 2 = “2–4 times a month”, 3 = “2–3 times a week”, 4 = “4 or more times a week”). In the second AUDIT-C question, the youth should rate how many drinks containing alcohol they consume on a typical day when they are drinking (five-step response format: 0 = “1–2”, 1 = “3–4”, 2 = “5–6”, 3 = “7–9”, 4 = “10 or more”). In the third AUDIT-C question, the adolescents were asked how often they consume six or more drinks on one occasion (five-step response format: 0 = “never”, 1 = “less than monthly”, 2 = “monthly”, 3 = “weekly”, 4 = “daily or almost daily”). An AUDIT-C sum value is formed by adding up the answers to all three questions and a higher sum value indicates a more pronounced problematic alcohol use. The reliability of the AUDIT-C was 0.72 in the investigated sample.

Problematic gaming in the past 12 months was assessed with the Internet Gaming Disorder Scale (IGDS, Lemmens et al. [37], we used the German version by Wartberg et al. [38]). The IGDS consists of nine questions with a binary answer format (0 = “no”, 1 = “yes”). An IGDS total value is calculated from the nine items. A higher total figure indicates a more pronounced problematic gaming. The reliability of the IGDS was 0.82 in the examined sample.

Problematic social media use in the last 12 months was measured with the Social Media Disorder Scale (SMDS, van den Eijnden et al. [10]). The SMDS also comprises nine items with a binary response format (0 = “no”, 1 = “yes”). An SMDS sum value can be calculated from the answers to the nine questions and a higher value indicates a more pronounced problematic social media use. The reliability of the SMDS was 0.83 in the surveyed sample.

To assess problematic Internet use, we utilized the Young Diagnostic Questionnaire (YDQ, Young [39], validation of the German version: Wartberg et al. [40]). The YDQ consists of eight items with a binary answer format (0 = “no”, 1 = “yes”) from which a sum value can be formed. A higher figure indicates a more pronounced problematic Internet use. The reliability of the YDQ was 0.70 in the investigated sample.

To assess adolescent mental health within the last six months, we applied the German adaption of the Reynolds Adolescent Adjustment Screening Inventory [41]: Screening psychischer Störungen im Jugendalter-II (SPS-J-II) [42]. The SPS-J-II consists of 32 questions (3-level response format: 0 = “never or almost never”, 1 = “sometimes”, 2 = “nearly all the time”). The questionnaire is divided into four scales assessing the frequency of adolescent antisocial behavior, anger control problems, emotional distress (combined measure of anxiety and depressiveness), and self-esteem problems. In each of the four scales, a higher sum value indicates a greater degree of adolescent psychopathological burden. We observed the following reliability coefficients (Cronbach’s α): antisocial behavior: α = 0.77, anger control problems: α = 0.79, emotional distress: α = 0.88 and self-esteem problems: α = 0.68.

To collect a parental rating of adolescent hyperactivity/inattention over the last six months, we utilized the scale hyperactivity/inattention of the well-established Strengths and Difficulties Questionnaire (SDQ) [43]. This SDQ subscale comprises five questions with a 3-level response format (0 = “not true”, 1 = “somewhat true”, 2 = “certainly true”). A higher total value in this scale indicates a higher level of adolescent hyperactivity/inattention. In our sample, the reliability coefficient of the SDQ scale was α = 0.75. Furthermore, socio-demographic characteristics (e.g., gender of the adolescent and age at the time of the survey) were collected.

### 2.4. Statistical Analyses

Overall, 319 adolescents had stated in the first question of the AUDIT-C [35] that they had never consumed alcohol in the last year. All these 319 cases did not have to answer the following two questions of the AUDIT-C and their AUDIT-C sum value was set to “0”. Similarly, 40 minors had reported that they had never played video games in the last twelve months. These 40 cases did not have to answer the IGDS questions [37] and their IGDS total value was set to “0”. Frequencies, mean values, standard deviations, reliability coefficients, correlations as well as multivariable linear regression analyses were calculated with SPSS version 25 (IBM, 2017, New York, NY, USA). First, bivariate correlation analyses were calculated. Subsequently, multivariable linear regression analyses were conducted (whereby all explanatory variables were simultaneously included in the regression models). In the four multivariable linear regression analyses problematic alcohol use, problematic gaming, problematic social media use, problematic Internet use were the dependent variable in one regression model each. In addition to gender, the different aspects of mental health (antisocial behavior, anger control problems, emotional distress, self-esteem problems, and hyperactivity/inattention) were utilized as explanatory variables.

## 3. Results

### 3.1. Descriptive Statistics

The sociodemographic characteristics of the total sample (*n* = 633) are presented in Table 1. In line with expectations (given the age of the sample and compulsory schooling in Germany), most of the adolescents interviewed were still attending school (93.5% of the total sample or 592 cases). For those youth who were still attending school, parents were asked to provide a prognosis for their future school leaving certificate (forecast). A total of 40 minors had already finished school (eight adolescents had achieved a graduation on a medium education level and another 32 adolescents on a high education level) and one girl did not go to school anymore (at the time of the survey she had no school leaving certificate).

### 3.2. Bivariate Correlation Analyses

Answers to RQ1, RQ2 and RQ3: In the bivariate correlation analyses, we observed statistically significant positive associations between problematic alcohol use and problematic gaming behavior, problematic social media use and problematic Internet use (see Table 2). Furthermore, statistically significant positive relations between problematic gaming behavior, problematic social media use and problematic Internet use were found (see Table 2).

### 3.3. Multiple Linear Regression Models

Answer to RQ4: In the multiple linear regression models problematic alcohol was associated with stronger antisocial behavior and lower anger control problems (see Table 3). Problematic gaming behavior was related to male gender and increased burden in all mental health aspects (antisocial behavior, anger control problems, emotional distress, self-esteem problems and hyperactivity/inattention) investigated. Problematic social media use was associated with female gender and higher levels of antisocial behavior, emotional distress, self-esteem problems and hyperactivity/inattention. Problematic Internet use was related to stronger antisocial behavior, higher emotional distress and more pronounced hyperactivity/inattention.

## 4. Discussion

In the present study, we investigated associations between problematic alcohol use and problematic use of the Internet and its specific applications (video games and social media) in adolescents. The first published findings already suggested relations between problematic alcohol use and problematic Internet use in adolescence [5,25,27,28,29]. We also found relationships between problematic alcohol use (operationalized via the established screening instrument AUDIT-C, which is recommended for use in adolescents, for example by Rumpf et al. [44]) and problematic Internet use (operationalized via the internationally frequently used YDQ [39]) in our sample of adolescents. Accordingly, our findings complement and confirm the international state of research on problematic alcohol use and problematic Internet use in adolescence.

Going beyond the current state of research, this study was the first to show empirically associations between problematic alcohol use and problematic gaming or problematic social media use in adolescence (a few findings have been reported from samples of adults). According to published empirical results (cross-sectional: e.g., Rosenkranz et al. [12], longitudinal: Wartberg et al. [13]), it does not seem advisable to transfer findings for a general problematic Internet use to a problematic use of specific online applications without further examination. In the context of the present study, it also seems helpful to distinguish between general problematic Internet use (in the cognitive-behavioral model of Davis [45] that would correspond to a “generalized pathological Internet use”) and the problematic use of specific applications (such as video games or social media, in Davis‘ model [45] that could be a “specific pathological Internet use” each).

Regarding the associations between problematic alcohol use and problematic gaming, there have so far been only two results in adults [30,31]. However, these studies revealed heterogeneous findings. While the study by Na et al. [30] showed a positive relation between problematic alcohol use and problematic gaming, Erevik et al. [31] observed a negative association. The present survey could not confirm Erevik et al.’s finding [31] that problematic gaming in adults functioned as a protective factor against problematic alcohol use. This result of the present investigation indicates a positive relationship between problematic alcohol use and problematic gaming in adolescence, but naturally requires verification in further empirical surveys. Concerning the relation between problematic alcohol use and problematic social media use, only one study in adults [32] is available. Lyvers et al. [32] reported a positive correlation between both problematic behavioral patterns, which we observed in the present study also for adolescents. Again, this finding needs to be verified in future studies.

According to Jessors Problem-Behavior Theory [3], problematic behavior in adolescence often does not occur in isolation but in combination (this applies not only to problematic alcohol use but also to the examined three problematic patterns of Internet use or its applications). However, a combined survey of substance-unrelated problematic behavioral patterns has so far only been carried out very rarely. Estévez et al. [46] investigated the importance of emotion regulation in a mixed sample of adolescents and young adults in Spain for various problematic behaviors related and unrelated to substance use. In the correlation table of all constructs studied, positive statistically significant correlations were found for alcohol abuse with both problematic Internet use (0.28) and video game addiction (0.13) and between problematic Internet use and video game addiction (0.35) [46]. In our sample of adolescents, we also observed statistically significant correlations between problematic alcohol use and problematic gaming (*r* = 0.12), problematic social media use (*r* = 0.14) and problematic Internet use (*r* = 0.13, see Table 2). However, it should be noted that, according to the classification of Cohen [47], these correlations were small, while the correlation between problematic gaming and problematic social media use was moderate (*r* = 0.45). There were large correlations between problematic Internet use and problematic gaming (*r* = 0.57) as well as between problematic Internet use and problematic social media use (*r* = 0.63). Correspondingly, the associations between problem behaviors without substance relations were much more pronounced than to the substance-related problem behavior. Large correlation coefficients between problematic Internet use and problematic gaming in adolescents have been reported before by Király et al. [48] with 0.59 (p. 752) and by van Rooij et al. [49] with 0.63 (p. 509).

With regard to the associations between the problematic behaviors and mental health aspects (as described previously in the Problem-Behavior Theory [3] and the I-PACE model [6]), we observed both similarities and differences. More pronounced antisocial behavior was related to problematic alcohol use, problematic gaming behavior, problematic social media use and problematic Internet use and therefore seems to be consistently relevant for all four problematic behavioral patterns. Whereas, emotional distress, self-esteem problems and hyperactivity/inattention were associated with substance-unrelated problematic behavior patterns, but not with problematic alcohol use. This finding is a further contribution to the question of whether problematic alcohol consumption is related to depressiveness, where the published empirical findings are still heterogeneous (e.g., [50]). More anger control problems were associated with problematic gaming, but surprisingly less pronounced anger control problems with problematic alcohol use. The finding that problematic alcohol use is associated with fewer anger control problems and more pronounced antisocial behavior is novel and needs further investigation. Whereas the relationship between a problematic alcohol use and more externalizing problems (e.g., conduct problems or antisocial behavior) is considered empirically well established.

The present survey has various limitations. The VEIF project does not examine a representative sample, because a higher percentage of youth with a higher risk of problematic use of digital media was included than in the general population (oversampling). This may limit the transferability of the results to the general population as well as to other age groups (e.g., adults). Findings of a cross-sectional analysis were reported. Therefore, no cause-effect relationships or conclusions that one behavior leads to another can be deduced between the characteristics examined (this would require a longitudinal design). In addition to the investigated behavior patterns, further problematic behavior patterns unrelated to substance use are conceivable (e.g., problematic gambling), which could also be relevant for problematic alcohol use in adolescence, but were not considered in the study design of the VEIF project. Therefore, we cannot provide a complete model to explain adolescent problematic alcohol use (too many relevant variables, such as substance use of the peers, were not collected).

## 5. Conclusions

Despite the limitations mentioned above, the present investigation revealed some interesting new findings. The empirical associations between problematic alcohol use and a problematic gaming and a problematic social media use among adolescents extended existing research. With regard to a common etiology and for the development of comprehensive prevention programs (e.g., life skills trainings), it seems quite promising to examine several problematic behavior patterns and their associations to mental health together. Therefore, the findings of the present survey can be used to develop or revise preventive measures. Furthermore, if there are indications of problem behaviors in youth related or unrelated to substance use, they should be screened for further substance-related or non-substance-related problematic behavior patterns as part of a comprehensive diagnostic procedure (e.g., before the start of an intervention measure in an outpatient or inpatient setting) in order to exclude or take them into account as comorbidities in treatment.

## Figures and Tables

**Table 1:** Sociodemographic characteristics of the sample.

Variable	Total Sample (*n* = 633) % or M (SD)
Gender	
Female	47.2%
Male	52.8%
Age ^a^	15.79 (0.96)
Achieved or prospective level of graduation ^b^	
Low-educational level	9.3%
Medium educational level	46.1%
High-educational level	44.5%

Note. ^a^ In years. ^b^ Prospective level: Forecast for all adolescents still attending school.

**Table 2:** Bivariate correlation analyses regarding the relationships of problematic alcohol use, problematic gaming behavior, problematic social media use and problematic Internet use in adolescents.

Variable	1	2	3	4
(1) Problematic alcohol use ^a^	–			
(2) Internet gaming disorder ^b^	0.12 **	–		
(3) Problematic social media use ^c^	0.14 ***	0.45 ***	–	
(4) Problematic Internet use ^d^	0.13 **	0.57 ***	0.63 ***	–

Note. ^a^ AUDIT-C sum value. ^b^ IGDS sum value. ^c^ SMDS sum value. ^d^ YDQ sum value; ** *p* < 0.01; *** *p* < 0.001.

**Table 3:** Multiple linear regression models regarding the associations between mental health aspects and problematic alcohol use, problematic gaming behavior, problematic social media use and problematic Internet use in adolescents.

Mental Health Aspects	Problematic Alcohol Use	Internet Gaming Disorder)	Problematic Social Media Use	Problematic Internet Use
	Standardized Beta Coefficients (95% CI)	Standardized Beta Coefficients (95% CI)	Standardized Beta Coefficients (95% CI)	Standardized Beta Coefficients (95% CI)
Gender ^a^	−0.01 (−0.08; 0.07)	−0.33 *** (−0.39; −0.27)	0.18 *** (0.12; 0.24)	−0.02 (−0.08; 0.05)
Antisocial behavior	0.52 *** (0.42; 0.62)	0.18 *** (0.11; 0.26)	0.26 *** (0.17; 0.34)	0.19 *** (0.10; 0.27)
Anger control problems	−0.15 ** (−0.25; −0.04)	0.13 *** (0.04; 0.21)	0.08 (−0.01; 0.17)	0.02 (−0.08; 0.11)
Emotional distress	−0.06 (−0.15; 0.03)	0.09 * (0.01; 0.16)	0.13 ** (0.05; 0.20)	0.28 *** (0.20; 0.36)
Self-esteem problems	0.02 (−0.06; 0.10)	0.10 ** (0.03; 0.16)	0.12 ** (0.05; 0.18)	0.06 (−0.01; 0.13)
Hyperactivity/inattention	0.01 (−0.09; 0.10)	0.22 *** (0.15; 0.29)	0.26 *** (0.19; 0.34)	0.26 *** (0.19; 0.33)

Note. ^a^ Coding: 0 = male, 1 = female. * *p* < 0.05; ** *p* < 0.01; *** *p* < 0.001.
